# Deeper but smaller: Higher-order interactions increase linear stability but shrink basins

**DOI:** 10.1126/sciadv.ado8049

**Published:** 2024-10-02

**Authors:** Yuanzhao Zhang, Per Sebastian Skardal, Federico Battiston, Giovanni Petri, Maxime Lucas

**Affiliations:** ^1^ Santa Fe Institute, Santa Fe, NM 87501, USA.; ^2^ Department of Mathematics, Trinity College, Hartford, CT 06106, USA.; ^3^ Department of Network and Data Science, Central European University, 1100 Vienna, Austria.; ^4^ NP Lab, Network Science Institute, Northeastern University London, London, UK.; ^5^ Department of Physics, Northeastern University, Boston, MA 02115, USA.; ^6^ CENTAI Institute, 10138 Torino, Italy.

## Abstract

A key challenge of nonlinear dynamics and network science is to understand how higher-order interactions influence collective dynamics. Although many studies have approached this question through linear stability analysis, less is known about how higher-order interactions shape the global organization of different states. Here, we shed light on this issue by analyzing the rich patterns supported by identical Kuramoto oscillators on hypergraphs. We show that higher-order interactions can have opposite effects on linear stability and basin stability: They stabilize twisted states (including full synchrony) by improving their linear stability, but also make them hard to find by markedly reducing their basin size. Our results highlight the importance of understanding higher-order interactions from both local and global perspectives.

## Teaser

Higher-order interactions affect collective dynamics differently when viewed from local and global perspectives.

## INTRODUCTION

Higher-order interactions are couplings that connect more than two units simultaneously and in a nonlinear way so that it cannot be decomposed into a linear combination of pairwise interactions (*1*–*6*). Such nonpairwise interactions are crucial in shaping complex dynamical processes such as contagion and cooperation in social networks (*7*–*13*), information processing in the brain (*14*–*18*), and synchronization in coupled oscillators (*19*–*23*). Understanding how they influence collective dynamics is thus essential. A variety of studies have approached this challenge from a linear stability perspective, which characterizes how states such as synchronization and consensus respond to small perturbations (*24*–*35*). However, little attention has been paid to basin stability (*36*), a global measure based on the size of basins of attraction, which dictates the system’s response to large perturbations (*37*–*41*).

Here, we provide a more complete picture of how higher-order interactions influence dynamical patterns, in terms of both linear and basin stability. We show that higher-order interactions can have opposite effects: They can increase the number of ordered states by making them linearly stable; at the same time, higher-order interactions also markedly shrink their attraction basins, effectively hiding them from detection. As a result, states such as full synchrony may be stable but unreachable from random initial conditions.

To demonstrate this point, we consider *n* identical phase oscillators coupled through both pairwise and triadic interactions

$$\begin{array}{cc} {\dot{\theta }}_{i}= & \omega +\frac{\sigma }{k_{i}^{\left(1\right)}}\sum_{j=1}^{n}‍A_{\mathrm{ij}}\text{sin}\left(\theta_{j}-\theta_{i}\right)\\ & \;\;+\frac{\sigma_{\Delta }}{2k_{i}^{\left(2\right)}}\sum_{j,k=1}^{n}‍B_{\mathrm{ijk}}\text{sin}\left(\theta_{j}+\theta_{k}-2\theta_{i}\right) \end{array}$$ (1)

Equation 1 is a generalization of the Kuramoto model (*42*), which can be derived exactly from the phase reduction of weakly coupled, nearly identical limit-cycle oscillators (*23*). In this sense, the Kuramoto dynamics represent a canonical model for a broad class of real-world systems exhibiting periodic oscillations. For example, Kuramoto dynamics with higher-order interactions have been used to analyze the collective dynamics of nanoelectromechanical oscillators observed in experiments (*20*). Here, θ*_i_* ∈ *S*^1^ represents the phase of oscillator *i* and ω is their common frequency. The adjacency tensors determine which oscillators interact: *A_ij_* = 1 if nodes *i* and *j* have a pairwise connection, and zero otherwise. Similarly, *B_ijk_* = 1 if and only if nodes *i*, *j*, and *k* are coupled through a triadic interaction. The coupling strengths are given by σ and σ_Δ_, respectively, and are normalized by $k_{i}^{\left(\ell \right)}$, the ℓth order degree of node *i*.

**Fig. 1.**
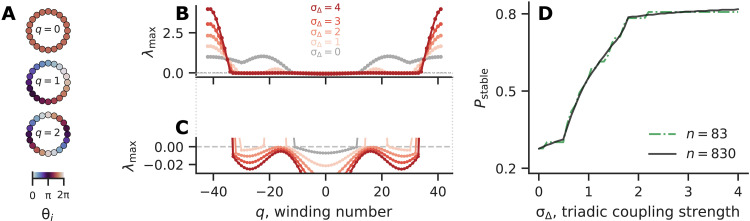
Higher-order interactions improve the linear stability of twisted states. (**A**) Example twisted states with different winding numbers, for *n* = 20. Full synchrony corresponds to *q* = 0. (**B**) Linear stability (measured by the largest transverse Lyapunov exponent λ_max_) of *q*-twisted states, for a range of triadic coupling strengths σ_Δ_. (**C**) Zoom-in view around λ_max_ = 0 showing which twisted states are stable (λ_max_ < 0). More twisted states become stable as σ_Δ_ is increased. (**D**) Fraction of twisted states that are stable as a function of σ_Δ_, for *n* = 83 and *n* = 830.

The case of pairwise coupling (σ_Δ_ = 0) has been studied in detail from both linear and basin stability perspectives. While full synchrony θ*_i_*(*t*) = θ*_j_*(*t*) ∀ *i*, *j*, *t* is always an attractor of Eq. 1, additional attractors can emerge when networks are not too dense (*43*–*46*). For ring networks, these attractors are twisted states and they emerge for link density below 0.68 (*47*). In a *q*-twisted state **θ**^(*q*)^, the phases make *q* full twists around the ring and satisfy $\theta_{k}^{\left(q\right)}=2\pi \mathrm{kq}/n+C$, where *q* is the winding number (c.f. Fig. 1A). In particular, the fully synchronized state corresponds to *q* = 0. For rings with nearest-neighbor couplings, the number of attractors grows linearly with *n*, since twisted states with up to *n*/4 twists are stable (*48*, *49*). A fruitful line of research aims to characterize the basins of the coexisting twisted states, which has revealed interesting scaling relations between basin size and winding number (*47*, *50*, *51*) as well as tentacle-like structures in the basins (*51*–*53*).

## RESULTS

### Linear stability analysis

Here, we focus on an analytically tractable case of Eq. 1 equipped with a simple ring structure

$$\begin{array}{cc} {\dot{\theta }}_{i} & =\frac{\sigma }{2r}\sum_{j=i-r}^{i+r}‍\text{sin}\left(\theta_{j}-\theta_{i}\right)\\ & +\frac{\sigma_{\Delta }}{2r\left(2r-1\right)}\sum_{j=i-r}^{i+r}‍\sum_{k=i-r}^{i+r}‍\text{sin}\left(\theta_{j}+\theta_{k}-2\theta_{i}\right) \end{array}$$ (2)

where *r* is the coupling range. For the triadic coupling, we require *i* ≠ *j* ≠ *k*, so that each triangle involves three distinct nodes. Notice that we have set ω = 0 by going into a rotating frame. Also note that we can always set σ = 1 by rescaling time, which we adopt in simulations throughout the paper. For demonstration purposes, all numerical results below are presented for Eq. 2 with *n* = 83 and *r* = 2, unless otherwise stated. The key findings remain qualitatively unchanged for other choices of the parameters. The choice of *n* = 83 simply follows the convention from earlier papers (*50*, *51*) and *r* = 2 is the smallest coupling range that allows nontrivial simplicial complexes (a popular class of hypergraphs that are heavily studied in the literature), which we investigate later in the paper.

Because of the rotational symmetry, twisted states are equilibria of Eq. 2. We first show that triadic interactions can stabilize twisted states far beyond what is possible with pairwise coupling. To analyze the linear stability of any fixed-point solution θ* of Eq. 1 (which includes Eq. 2 as a special case), we can write down the Jacobian ***J***(θ*) = ***J***^(1)^(θ*) + ***J***^(2)^(θ*), where

$$\begin{array}{c} J_{\mathrm{ij}}^{\left(1\right)}\left(\theta^{*}\right)=\frac{\sigma }{k_{i}^{\left(1\right)}}A_{\mathrm{ij}}\text{cos}\left(\theta_{j}^{*}-\theta_{i}^{*}\right),\\ J_{\mathrm{ij}}^{\left(2\right)}\left(\theta^{*}\right)=\frac{\sigma_{\Delta }}{k_{i}^{\left(2\right)}}\sum_{k=1}^{n}‍B_{\mathrm{ijk}}\text{cos}\left(\theta_{j}^{*}+\theta_{k}^{*}-2\theta_{i}^{*}\right) \end{array}$$ (3)

for *i* ≠ *j* and $J_{\mathrm{ii}}^{\left(\ell \right)}=\underset{j\neq i}{-\sum_{j=1}^{n}J_{\mathrm{ij}}^{\left(\ell \right)}}$. For the rotationally symmetric topology considered in Eq. 2, the Jacobian is a symmetric circulant matrix of the form

$$J=\left[\begin{array}{ccccccc} J_{0} & J_{1} & J_{2} & \cdots & J_{3} & J_{2} & J_{1}\\ J_{1} & J_{0} & J_{1} & \cdots & J_{4} & J_{3} & J_{2}\\ \vdots & \vdots & \vdots & \ddots & \vdots & \vdots & \vdots \\ J_{2} & J_{3} & J_{4} & \cdots & J_{1} & J_{0} & J_{1}\\ J_{1} & J_{2} & J_{3} & \cdots & J_{2} & J_{1} & J_{0} \end{array}\right]$$ (4)

We know that the normalized eigenvectors of a circulant matrix are the Fourier modes and the eigenvalues of ***J*** are given by

$$\lambda_{p}\left(J\right)=\sum_{s=0}^{n-1}‍J_{s}e^{2\pi \mathrm{ips}/n},\;0\leq p\leq n-1$$ (5)

where *J_s_* = *J*_*n*−*s*_ for $\left\lfloor n/2\right\rfloor$ < *s* < *n* (*54*). This implies that λ*_p_* are all real. Moreover, λ_0_ is always equal to 0, and it represents the mode for which all oscillators are perturbed by the same amount. For any *q*-twisted state, **θ**^(*q*)^, *J_s_* is given by

$$\begin{array}{cc} J_{s}= & \frac{\sigma }{2r}\text{cos}\left(\frac{2\pi q}{n}s\right)+\frac{\sigma_{\Delta }}{r\left(2r-1\right)}\sum_{k=-r}^{r}‍\text{cos}\left[\frac{2\pi q}{n}\left(s+k\right)\right]\\ & -\frac{\sigma_{\Delta }}{r\left(2r-1\right)}\sum_{j=1}^{2}‍\text{cos}\left(\frac{2\pi q}{n}\mathrm{js}\right)\;\text{for}\;0<s\leq r,\\ J_{s}= & 0\;\text{for}\;s>r,\\ J_{0}= & -2\sum_{s=1}^{r}‍J_{s} \end{array}$$

For example, for *r* = 2,

$$\begin{array}{c} J_{1}=\frac{\sigma }{4}\text{cos}\left(\frac{2\pi q}{n}\right)+\frac{\sigma_{\Delta }}{6}\left[1+\text{cos}\left(\frac{2\pi q}{n}\right)+\text{cos}\left(\frac{6\pi q}{n}\right)\right],\\ J_{2}=\frac{\sigma }{4}\text{cos}\left(\frac{4\pi q}{n}\right)+\frac{\sigma_{\Delta }}{6}\left[1+\text{cos}\left(\frac{2\pi q}{n}\right)+\text{cos}\left(\frac{6\pi q}{n}\right)\right] \end{array}$$

Plugging the formula for *J_s_* into Eq. 5, we can analytically obtain the spectrum of the Jacobian for any *q*-twisted state and any coupling range *r*.

In Fig. 1 (B and C), we show the linear stability of twisted states, measured by the largest Lyapunov exponent transverse to the synchronization manifold, λ_max_ = max {λ_1_, λ_2_, …, λ_*n*−1_}, as a function of the winding number *q*. First, note that we show *n* values of *q* because, by definition, there are only *n* distinct twisted states: *q* → *q* + *n* simply adds 2π to all phases, leaving them unchanged. We consider $-\left\lfloor \frac{n}{2}\right\rfloor \leq q\leq \left\lfloor \frac{n}{2}\right\rfloor$; because twisted states with winding numbers −*q* and *n-q* are the same, one could equivalently consider the interval 0 ≤ *q* ≤ *n*. Second, note that the plot is symmetric with respect to *q* = 0. This follows from the fact that twisted states with winding numbers *q* and −*q* are the same up to the reversal symmetry **θ** →−**θ**.

Because of this symmetry, below we only need to describe states with positive *q*. At σ_Δ_ = 0, the stability curve has three peaks, and λ_max_ becomes positive for *q* > 11. Thus, pairwise coupling in Eq. 2 cannot support stable twisted states with more than 11 twists (for *n* = 83 and *r* = 2). For systems with strong triadic couplings, two of the peaks are flattened. As a result, a lot more twisted states become stable. For example, all twisted states up to 33 twists are stable for σ_Δ_ = 4. For intermediate σ_Δ_, because λ_max_ is a nonmonotonic function of *q*, winding numbers on disjoint intervals can become stabilized. For instance, at σ_Δ_ = 1, twisted states are stable for *q* ≤ 14, become unstable for 14 < *q* < 23, then become stable again for 23 ≤ *q* ≤ 30 (see Fig. 1C for details). Another interesting phenomenon to note is that the gradient of λ_max_ becomes much steeper upon crossing the *x* axis from below. This is caused by the switch of the dominant eigenvector, which we further elaborate in fig. S1.

Figure 1D shows the fraction of stable twisted states *P*_stable_ as a function of σ_Δ_, which further emphasizes the dramatic number of twisted states stabilized by triadic interactions. We see that *P*_stable_ is monotonically increasing with σ_Δ_, and that one can easily go from less than 30% of stable twisted states to over 80% stable by adding triadic interactions. We also show the same curve for a larger system with *n* = 830, which is basically a smoother version of *n* = 83 (since there are a lot more twisted states for *n* = 830). Our results echo the recent findings in (*55*), which showed that higher-order interactions can stabilize twisted states in graphons.

### Basin stability analysis

**Fig. 2.**
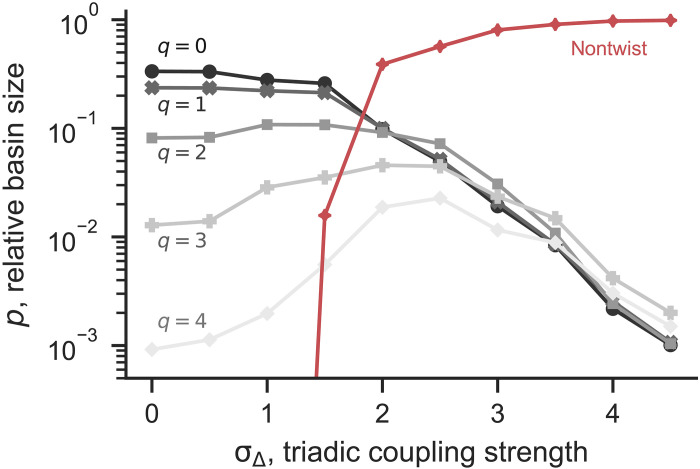
Higher-order interactions decrease the basin stability of twisted states. We show *p*, the relative basin size, as a function of the triadic coupling strength σ_Δ_. We estimated *p* by simulations starting from 10^5^ random initial conditions. Each line represents a *q*-twisted state, except the red line, which represents attractors that are not twisted states. The relative basin size of nontwisted states quickly approaches 1 as σ_Δ_ is increased. We only show *q* ≥ 0 due to the symmetry between *q* and −*q*.

Next, we switch to the basin stability perspective. Figure 2 shows how the relative basin size *p* of the twisted states changes with the triadic coupling strength σ_Δ_. We compute the basin size relative to the full state space (which is compact) by simulating the dynamics starting from 10^5^ random initial conditions and counting the proportion of those that converge to a given state. For σ_Δ_ = 0, twisted states (including full synchrony) are the only stable states and thus take up the entire state space (*56*) (see section S1 for details). States with fewer twists (smaller *q*) have a larger basin size. In particular, full synchrony attracts the most initial conditions. Now, for small σ_Δ_, triadic interactions are affecting twisted states unequally: The basins for small *q* shrink, whereas those for large *q* expand. As σ_Δ_ is further increased, the basins for the nontwisted states appear and quickly become dominant, whereas the basins of the twisted states all shrink and become comparable in size. Note that among the twisted states, full synchrony does not have the largest basin anymore; the twisted state with the largest basin has more twists as σ_Δ_ is increased.

**Fig. 3.**
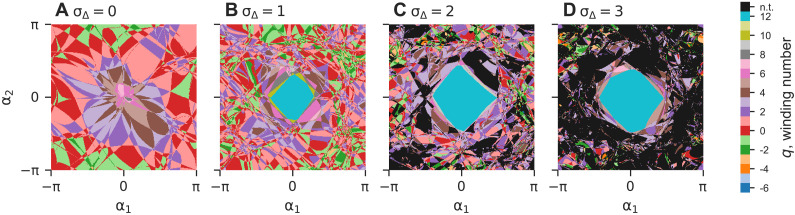
Higher-order interactions stabilize twisted states but shrink their basins. Two-dimensional (2D) slices of the state space (centered around the twisted state with *q* = 12) showing how basins change as σ_Δ_ is increased. The basins of the twisted states are colored according to their winding number *q*, and the basins of all other states are colored black. (**A**) For σ_Δ_ = 0, the twisted state with *q* = 12 is unstable, and all points converge to a twisted state with a lower winding number. (**B**) For σ_Δ_ = 1, all attractors are still twisted states. Moreover, *q* = 12 becomes stable, which creates the cyan basin at the center. (**C** and **D**) For stronger triadic interactions (σ_Δ_ = 2 and 3), the *q* = 12 basin expands, but nontwisted states also start to appear and quickly become dominant. Although the basin for *q* = 12 looks substantial on the 2D slices, due to the high-dimensional nature of the state space, it would be almost impossible to reach from random initial conditions.

Figure 3 further illustrates the opposite effects of higher-order interactions on linear stability and basin stability by visualizing the morphology of basins as σ_Δ_ is increased. Specifically, we examine a random two-dimensional (2D) slice of the state space, spanned by **θ**_0_ + α_1_***P***_1_ + α_2_***P***_2_, α*_i_* ∈ (−π, π]. Here, ***P***_1_ and ***P***_2_ are *n*-dimensional binary orientation vectors in which $\left\lfloor n/2\right\rfloor$ randomly selected components are 1 and the rest of the components are 0. We set the origin to be the twisted state with *q* = 12, **θ**_0_ = **θ**^(12)^, which we know from Fig. 1 is unstable when σ_Δ_ = 0. Thus, we can only see basins for *q* between −3 and 7 in Fig. 3A. Adding triadic interactions stabilizes *q* = 12, so we see its basin emerge in Fig. 3B when σ_Δ_ is set to 1. For larger σ_Δ_ shown in Fig. 3 (C and D), the reduction in basin stability for twisted states becomes apparent. Despite their substantially improved linear stability, the basins for twisted states (as a group) rapidly shrink as more and more points get absorbed into the basin for nontwisted states (colored black), making twisted states hard to reach from random initial conditions.

**Fig. 4.**
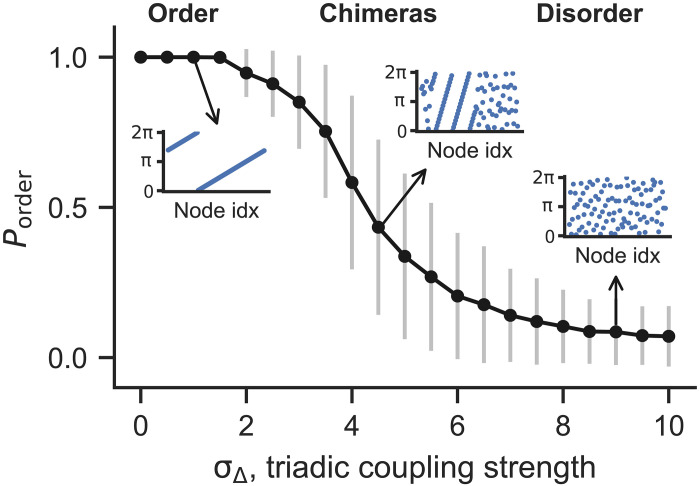
Chimeras bridge the transition from order to disorder as triadic interactions become stronger. As we increase σ_Δ_, there is a smooth transition from order (twisted states) to chimeras, and then to disorder. We monitor the transition by computing the portion of ordered oscillators *P*_order_ in the final state, averaged over 1000 simulations from random initial conditions for each σ_Δ_. The error bars represent SDs and the insets show typical attractors reached from random initial conditions.

The natural next question is: What are those nontwisted states created by higher-order interactions? Figure 4 shows that they consist of chimera states with increasingly large disordered domains as triadic couplings become stronger. Here, *P*_order_ measures the portion of oscillators that are ordered. Twisted states correspond to *P*_order_ = 1 and disordered states correspond to *P*_order_ ≈ 0, whereas chimera states have *P*_order_ between 0 and 1. For final states reached from random initial conditions, the average *P*_order_ decreases gradually from 1 to 0 as σ_Δ_ is increased.

We classify oscillators as (dis)ordered by calculating the local order parameters

$$O_{j}=\frac{1}{2r+1}\sum_{k=j-r}^{j+r}‍e^{i\theta_{k}}$$ (6)

for *j* = 1, …, *n*. We classify oscillator *j* as disordered if ∣*O_j_*∣ < 0.85. The value of 0.85 is large enough to ensure *P*_order_ ≃ 0 for large σ_Δ_, which matches visual inspection of the states. Although this analysis is phenomenological in nature, the twisted states would have ∣*O_j_*∣ ≈ 1 if the winding numbers and coupling ranges are small, which are satisfied for the systems in Fig. 4.

The insets show typical attractors for different values of σ_Δ_. Despite the fact that more and more twisted states become linearly stable for larger σ_Δ_, they are increasingly unlikely to be observed from random initial conditions. Instead, the state space is dominated by the basins for chimera states (intermediate σ_Δ_) or disordered states (large σ_Δ_). This is consistent with recent results showing that higher-order interactions promote chimera states in simplicial complexes (*57*). Similar states have also been observed recently in a continuous-space system (*58*). We note that the exact appearance of chimeras or disordered states can vary for different coupling ranges or coupling structures. However, the order-chimera-disorder transition described here is a robust phenomenon.

We also note that here, the disorder is only in space, not in time; the patterns either remain frozen over time (fixed points) or rotate uniformly with a constant speed (periodic orbits). In particular, all oscillators are phase locked. This is in contrast to traditional chimeras, for which the disordered oscillators are not frequency synchronized and their relative phases change over time. In fig. S2, we show the statistics of the effective frequency for different initial conditions and under different coupling strengths σ_Δ_. For small σ_Δ_, the effective frequency is always 0, but nonzero frequency can emerge for σ_Δ_ ≥ 2. This is consistent with recent results showing that twisted states can undergo Hopf bifurcations as σ_Δ_ is increased (*59*), which also consider the effect of higher-order interactions on Kuramoto oscillators but differ from our systems in crucial details.

### Other coupling structures

**Fig. 5.**
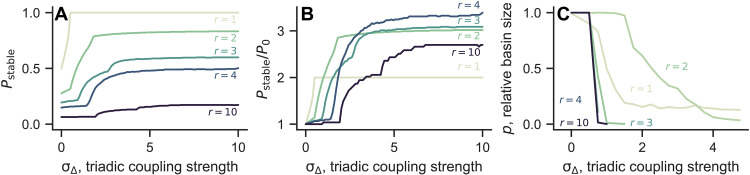
Higher-order interactions increase linear stability while decreasing basin stability for hypergraph rings with different coupling ranges. (**A**) Fraction of twisted states that are stable as a function of σ_Δ_, calculated with *n* = 830. (**B**) Same data as in (A), but shown as the ratio between the number of stable twisted states at nonzero σ_Δ_ and σ_Δ_ = 0. (**C**) Relative basin size of all twisted states combined as a function of σ_Δ_. For any given *r*, twisted states become more difficult to find as the triadic couplings become stronger. The basin sizes are estimated by simulating *n* = 83 oscillators from 10^3^ random initial conditions.

The above results for *r* = 2 remain qualitatively unchanged for other coupling ranges *r*. Figure 5 shows the opposite behavior of linear and basin stability for a wide range of *r*. For *r* = 1, 50% of the twisted states are stable under purely pairwise coupling. As σ_Δ_ is increased, all twisted states quickly become stabilized. As the coupling structure becomes more nonlocal (larger *r*), fewer twisted states are stable at σ_Δ_ = 0. By introducing triadic couplings, one can always have at least twice as many stable twisted states. For *r* ≥ 3, we also observed the appearance of two-cluster states, that is, with oscillators split (potentially unequally) into two π-separated clusters [described, e.g., in (*60*–*62*)], as shown in figs. S3 and S4.

Aside from the generalization of Kuramoto models introduced in Eq. 2, there are several other natural ways to introduce triadic interactions. For example, we can turn the (pairwise) ring network into a simplicial (flag) complex by filling all pairwise triangles. This implies that *B_ijk_* = 1 if and only if *i* ≠ *j* ≠ *k*, and all three pairs are within distance *r*: ∣(*i* − *j*) mod *n*∣ ≤ *r*, ∣(*i* − *k*) mod *n*∣ ≤ *r*, and ∣(*j* − *k*) mod *n*∣ ≤ *r*. In other words, *B_ijk_* = *A_ij_A_ik_A_jk_*. In comparison, the topologies considered in Eq. 2 do not require ∣(*j* − *k*) mod *n*∣ ≤ *r* for *B_ijk_* = 1. This can be expressed equivalently as *B_ijk_* = *A_ij_A_ik_*. As shown in fig. S5, the main results presented above for ring hypergraphs remain valid for simplicial complexes.

**Fig. 6.**
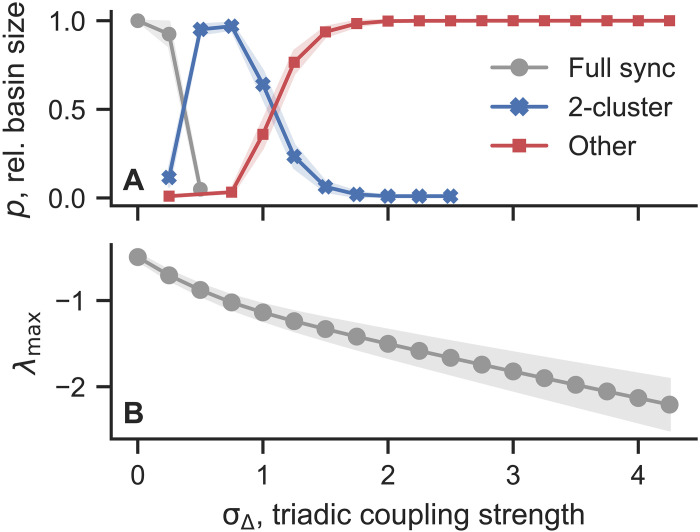
Synchronization becomes linearly more stable but harder to reach in random hypergraphs. We show (**A**) the relative basin size of full synchrony (zero-twisted), two-cluster, and other (nontwisted) states, and (**B**) the maximum transverse Lyapunov exponent for full synchrony. Results show the average over 20 random hypergraph realizations with *n* = 83 nodes (with 100 initial conditions each). The shaded areas indicate one SD.

Last, we demonstrate that a similar phenomenon persists in more irregular structures using random hypergraphs. A random hypergraph is built similarly to an Erdős-Rényi random graph: We add a hyperedge between any two nodes with probability *p*_1_ and between any three nodes with probability *p*_2_. We set *p_d_* = 20/*n^d^*, for *d* = 1,2. For random hypergraphs, the only twisted state that can be an attractor is full synchrony (*q* = 0). Similar to what we found above for more regular structures, in random hypergraphs triadic interactions make full synchrony linearly more stable (*24*, *33*) but its basin of attraction shrinks dramatically in favor of two-cluster states, for σ_Δ_ up to around 1.5, and then more disordered states for stronger triadic coupling strengths (Fig. 6).

## DISCUSSION

Here, we showed that higher-order interactions can make basins deeper but smaller; attractors become linearly more stable but at the same time are harder to find due to their basins shrinking markedly. We demonstrated this phenomenon for Kuramoto dynamics with a wide range of coupling structures (ring hypergraphs with different coupling ranges, ring simplicial complexes, and random hypergraphs). We were able to characterize the linear stability of all twisted states analytically under these coupling structures. For basin stability, our systematic numerical simulations revealed interesting global features of the dynamics as σ_Δ_ is increased. In particular, the basins of twisted states become deeper but smaller due to the proliferation of new attracting states. We further characterized these new states introduced by higher-order interactions that compete with twisted states, which manifest as two-cluster, chimera, or disordered states depending on the ratio between σ and σ_Δ_.

Deeper but smaller basins induced by higher-order interactions can confer functional advantages to some biological systems. For instance, for the brain to function optimally (*63*), the attractors should have high linear stability so the brain can quickly return to the current state when subject to small perturbations or noise. At the same time, we also want the brain to be nimble and able to transition among different states efficiently (e.g., during computation and information processing), which can be achieved by having small basins.

Why do the basins of twisted states shrink as higher-order couplings are introduced? First, we note that, unlike their pairwise counterparts, Kuramoto systems with nonpairwise interactions are generally not gradient systems (*59*). This provides the freedom for Eqs. 1 and 2 to undergo Hopf bifurcations as σ_Δ_ is increased. Böttche *et al.* (*64*) showed recently that anticorrelation between linear stability and basin stability often emerges for dynamical systems that undergo consecutive Hopf bifurcations, offering a potential mechanism for higher-order interactions to shrink basins. More generally, in a compact phase space, as more attractors are created, the average basin size would decrease. In our case, the new states that emerge are more disordered than twisted states and they hold enormous entropic advantages (there are many more possible disordered configurations than ordered ones). Even two-cluster states, which appear ordered on the surface, have many more configurations than twisted states; the oscillators can be divided between the two clusters in 2*^n^* different ways (*61*).

Does extensive multistability emerge naturally from generic higher-order interactions regardless of details about the dynamics and couplings? Such phenomena have been observed under many different settings (*57*, *61*, *65*, *66*). For Eq. 1 with all-to-all coupling, it was shown previously that the coupling function sin(θ*_j_* + θ*_k_* − 2θ*_i_*) introduces a higher-order harmonic and nonlinear dependence on the order parameter in the mean-field description, which create additional nonlinearity and extensive multistability in the self-consistent equations for the order parameters (*67*, *68*). The same is true for a different coupling function, sin(2θ*_j_* − θ*_k_* − θ*_i_*): Using the Ott-Antonsen ansatz (*69*), it was found that higher-order interactions give rise to added nonlinearity in the reduced equations that describe the macroscopic system dynamics (*70*). Moreover, when deviating from all-to-all coupling (such as the local couplings considered here), we expect the nontrivial coupling structure could introduce additional nonlinearity into the macroscopic equations, further increasing multistability.

It is possible that deeper but smaller basins may not be the exclusive results of higher-order interactions. Can other forms of nonlinear coupling functions create similar effects? If so, what are the properties required of those nonlinearities? For example, another way to add nonlinearity to the Kuramoto model is through higher-harmonic coupling functions such as sin(2θ*_j_* − 2θ*_i_*) (*62*, *71*). In our preliminary tests, however, we found that they do not stabilize more twisted states. The systematic exploration of the link between nonlinearity and stability (both local and global) is an important and open question, which we leave for future works.

In conclusion, the prevalence of anticorrelation between linear stability and basin stability warrants a more nuanced and comprehensive approach when considering collective dynamics on hypergraphs and simplicial complexes. Understanding the global organization of attractors and saddles in the presence of nonpairwise couplings is crucial to the prediction and control of complex systems such as ecological communities and neuronal populations. We hope that this work will stimulate future endeavors to understand the effects of higher-order interactions from both local and global perspectives.

## Supplementary Materials

### This PDF file includes:

Sections S1 to S5 Figs. S1 to S5
