# Supplementary material for: Deeper but smaller: Higher-order interactions increase linear stability but shrink basins
Source: Sci Adv. 2024 Oct 2;10(40):eado8049. doi: 10.1126/sciadv.ado8049 (PMC11446277; doi:10.1126/sciadv.ado8049)
Supplement: Supplementary file 1 — Sections S1 to S5 Figs. S1 to S5 [file sciadv.ado8049_sm.pdf]

Science Advances
AAAS

Supplementary Materials for

Deeper but smaller: Higher-order interactions increase linear stability but shrink basins

Yuanzhao Zhang *et al.*

Corresponding author: Yuanzhao Zhang, yzhang@santafe.edu; Maxime Lucas, maxime.lucas.work@gmail.com

Sci. Adv. **10**, eado8049 (2024)
DOI: 10.1126/sciadv.ado8049

This PDF file includes:

Sections S1 to S5
Figs. S1 to S5

S1 Global stability of twisted states on ring networks

In pairwise ring networks of identical Kuramoto oscillators, all attractors are twisted states. The basic arguments are as follows: Identical Kuramoto oscillators on symmetric networks are described by the gradient of a smooth potential on the n-torus. Because gradient dynamics on a compact manifold can only have fixed points as attractors, we can rule out periodic or chaotic solutions. For fixed points, because of the ring structure, the steady-state phase differences between neighboring oscillators have to be either δ or $\pi - \delta$, with a $\delta = 2\pi q/n$ that is independent of the node indices. Through linear stability analysis (or simply by using the Gershgorin circle theorem), one can show that the only fixed points that are stable are those with all phase differences $\theta_{i+1} - \theta_i$ equal to δ. This then establishes that twisted states are the only attractors.

S2 Crossing of eigenmodes

In Fig. 1, the steepness of the $\lambda_{\max}$ curves changed dramatically at the instability transition because the dominant eigenvector of the Jacobian changed. The basic intuition is that there are eigenmodes that oscillate much more "wildly" than others. In Fig. S1 below, each curve represents an eigenvalue. The orange curve is the most "tame" curve and usually gives the largest transverse Lyapunov exponent $\lambda_{\max}$ when all the eigenvalues are negative. However, around $\lambda = 0$, it gets overtaken by other eigenmodes with much steeper gradients. This explains why $\lambda_{\max}$ suddenly shoots up rapidly after crossing the x-axis from below.

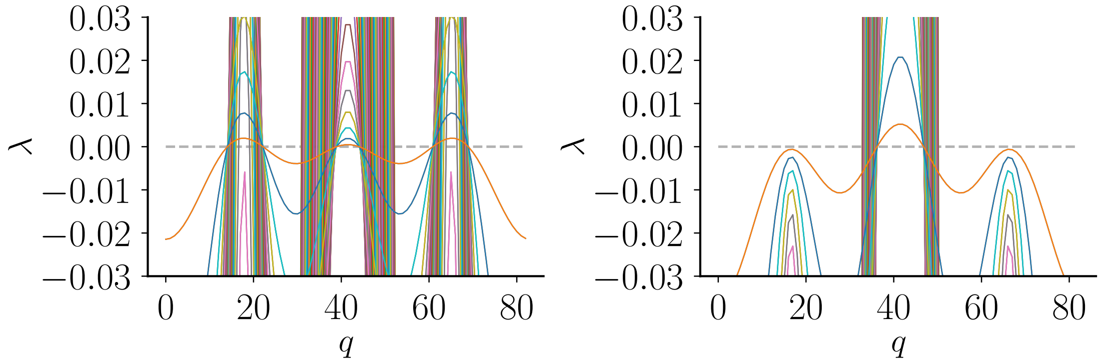

Supplementary Figure S1: Full spectrum of the Jacobian for the system in Fig. 1 with $\sigma_\Delta = 1$ (left) and $\sigma_\Delta = 2$ (right).

S3 Frequency analysis of chimera states

For the states shown in Fig. 4, the rotation speed is always constant and identical across all oscillators, regardless of whether they are spatially ordered, disordered, or a mixture of both. Figure S2 shows the effective frequency Ω for different initial conditions and under different coupling strengths σ_Δ. Here, Ω is computed as an average over all oscillators and over time. However, this is not crucial as all oscillators have the same frequency and are moving with constant speed.

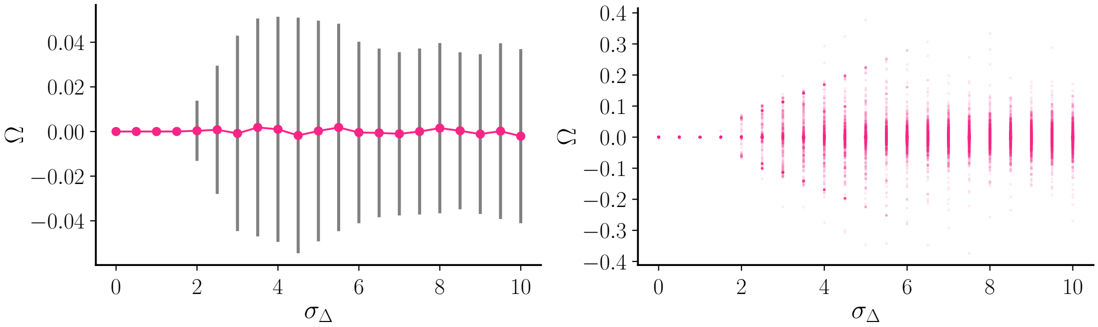

Supplementary Figure S2: Effective frequency Ω of the oscillators for Eq. (2) with $n = 83$ and $r = 2$. At each value of σ_Δ, 1000 initial conditions were simulated. The left panel shows the average over initial conditions and the corresponding standard deviations. The right panel shows a scatter plot of the effective frequency for each initial condition.

S4 Ring hypergraphs with different coupling ranges

Here, we consider the dynamics on ring hypergraphs from Eq. (2) and vary the coupling range r. In Fig. S3, we show the relative basin sizes of the twisted states, the 2-cluster states, and the other states remaining. Here, we are mainly interested in how much space each category of states takes. Therefore, the basin size for the twisted states is aggregated over all twisted states, and that of the 2-cluster states is aggregated over all possible configurations of the two clusters. Consistent with our results in the main text, the basin size associated with twisted states drops for larger triadic coupling strengths while the basin associated with other states takes most of the space. An additional type of state also appears—as described in the main text, in these 2-cluster states, oscillators are split (usually unequally) into two π-separated clusters. Figure S4 shows the same data differently: Each panel corresponds to a single value of r and shows one curve per category of states, more similarly to Fig. 2.

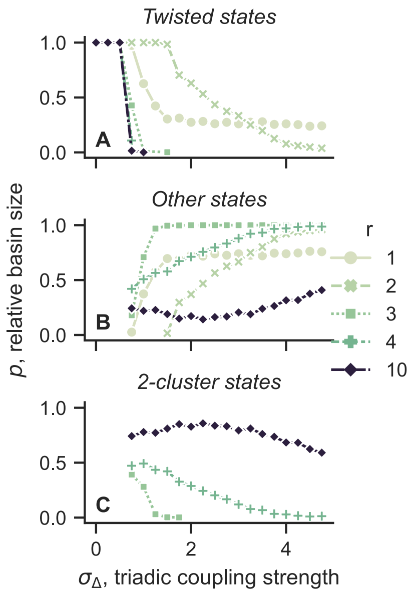

Supplementary Figure S3: **Extension of Fig. 5c for ring hypergraphs with different coupling ranges** r**.** Here we also show relative basin sizes of (A) twisted states, (B) other states, and (C) 2-cluster states. Two-cluster states appear for $r \geq 3$.

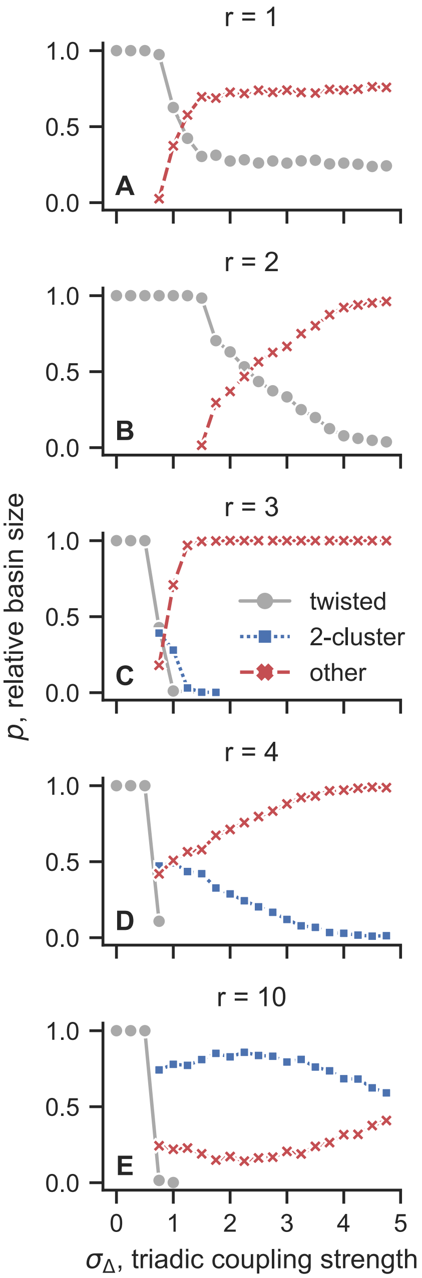

Supplementary Figure S4: **Same data as in Fig. S3 for ring hypergraphs with different couping ranges.** Here we show the basin sizes per value of r.

S5 Ring simplicial complexes

For simplicial complexes, we consider systems described by the following equations:

$$\dot{\theta}_i = \frac{\sigma}{2r}\sum_{j=i-r}^{i+r}\sin(\theta_j-\theta_i) + \frac{\sigma_\Delta}{3r(r-1)}\sum_{\substack{0<|k-i|\le r\\ 0<|j-i|\le r\\ 0<|j-k|\le r}}\sin(\theta_j+\theta_k-2\theta_i), \quad i=1,\dots,n. \tag{S1}$$

It is easy to verify that the coupling structure forms a simplicial complex for any coupling range r.

For simplicial complexes with coupling range r, we have

$$J_s = \frac{\sigma}{2r}\cos(\frac{2\pi q}{n}s) + \frac{2\sigma_\Delta}{3r(r-1)}\sum_{k=s-r}^{r}\cos(\frac{2\pi q}{n}(s+k)) - \frac{2\sigma_\Delta}{3r(r-1)}\sum_{j=1}^{2}\cos(\frac{2\pi q}{n}js) \tag{S2}$$

for $0<s\le r$. Specifically, for $r=2$ we have

$$\begin{aligned} J_1 &= \frac{\sigma}{4}\cos(\frac{2\pi q}{n}) + \frac{\sigma_\Delta}{3}\left[1+\cos(\frac{6\pi q}{n})\right], \\ J_2 &= \frac{\sigma}{4}\cos(\frac{4\pi q}{n}) + \frac{\sigma_\Delta}{3}\cos(\frac{6\pi q}{n}). \end{aligned} \tag{S3}$$

We show the results for this structure in Fig. S5.

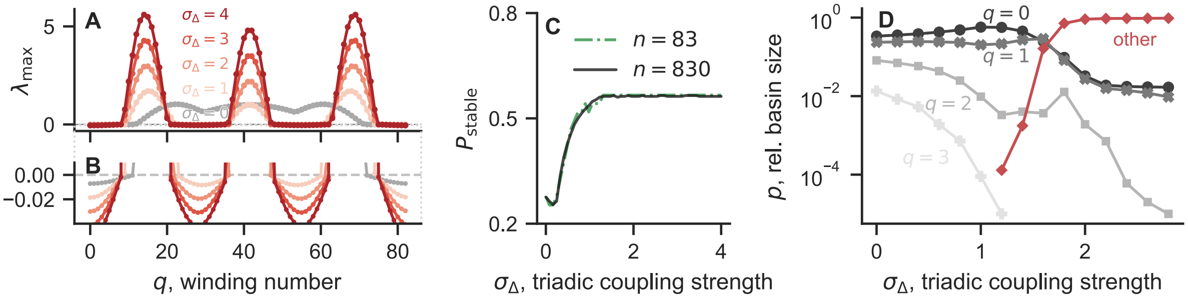

Supplementary Figure S5: **Analog of Figs. 1 and 2 for ring simplicial complexes.** Here, we show detailed results for the coupling range $r = 2$, but they remain qualitatively unchanged for larger r.
